# Tripartite Interactions in Biocontrol: Insights for Developing Yeast-Based Strategies

**DOI:** 10.3390/microorganisms13102307

**Published:** 2025-10-05

**Authors:** Anuruddha Karunarathna, Dulanjalee Lakmali Harishchandra, Sukanya Haituk, Saruta Arayapichart, Thitima Wongwan, Ratchadawan Cheewangkoon

**Affiliations:** 1 Office of the Research Administration, Chiang Mai University, Chiang Mai 50200, Thailand; kapuduwavitharanag.k@cmu.ac.th (A.K.); dulanjalee.h@cmu.ac.th (D.L.H.); sukanya.h@cmu.ac.th (S.H.); 2 Department of Entomology and Plant Pathology, Faculty of Agriculture, Chiang Mai University, Chiang Mai 50200, Thailand; saruta_a@cmu.ac.th (S.A.); thitima.wongwan@cmu.ac.th (T.W.)

**Keywords:** environmental health, omics tools, pesticide reduction, plant–pathogen–BCA interactions, sustainable agriculture, transcriptomics

## Abstract

Conventional plant disease management primarily depends on chemical pesticides. However, with the rising concerns related to human health, environmental sustainability, and the emergence of resistant pathogens, biocontrol agents (BCAs) have gained more attention as eco-friendly alternatives. Among the potential biocontrol agents, yeasts stand out due to their safety, adaptability, and diverse antagonistic mechanisms, ranging from competition and enzyme secretion to volatile compound production and immunity induction. Despite their potential, yeast-based BCAs face limitations in field efficacy, regulation, and an incomplete understanding of their molecular interactions. Most current studies focus on simple, pairwise interactions, overlooking the complexity of agroecosystems, where plants, pathogens, and BCAs interact within broader microbial communities. This review addresses the importance of understanding tripartite interactions among plants, pathogens, and yeasts, supported by integrated transcriptomic and comparative genomic approaches, as well as meticulous observations of phenotypic expressions to uncover strain-specific defense mechanisms and mode of action. By referring to well-studied models like *Blumeria graminis* f.sp. *hordei*–*Hordeum vulgare*–*Pseudozyma flocculosa* and *Trichoderma* tripartite systems, we highlight the underexplored potential of yeasts to modulate plant immunity and influence pathogen behavior through complex molecular crosstalk. Bridging these knowledge gaps through integrating proteomic, metabolomic, and transcriptomic analyses, we can better harness yeasts in sustainable and targeted biocontrol strategies.

## 1. Introduction

Conventional plant disease control techniques involve the application of synthetic chemicals. Given their negative effects on human health, impact on the environment, and pathogen resistance, their efficiency is questionable [1]. Hence, the elimination of or reduction in chemical pesticides is highly desirable in modern agriculture. With all these concerns in mind, biocontrol agents (BCAs) are becoming the most potent approach. Successful biocontrol occurs through successful interactions between plants, biocontrol agents, and pathogens [2]. Microbes inhabiting various plant tissues (leaves, roots, flowers, seeds) play a pivotal role in plant health [3]. In certain cases, these microorganisms show certain biocontrol properties, and they become potent biocontrol agents. So far, many microorganisms (fungi, bacteria, and yeasts) have been investigated as biocontrol agents, and several BCAs have been used commercially (*Trichoderma harzianum* [4], *Metschnikowia fructicola* [5], *Bacillus subtilis* [6]).

Among biocontrol agents, yeast shows some unique characteristics, such as being ubiquitous microorganisms that are safe, easy to cultivate, and adaptable to various environments. Most importantly, yeasts became a hot topic as a BCA in recent years given their ability to antagonize a range of plant pathogens, offering an eco-friendly alternative to chemical pesticides [2]. Yeasts can outcompete pathogens by rapid colonization of plant surfaces and consuming available nutrients, thereby preventing pathogen establishment [7]. This leads to competition for nutrients and space. Some yeasts inhibit the growth and spread of pathogenic fungi by producing enzymes that degrade the cell walls of pathogenic fungi [8]. Further, certain yeast strains produce killer toxins or mycocins that are lethal to specific pathogens, providing targeted biocontrol. Interestingly, certain yeasts emit growth-suppressing volatile organic compounds (VOCs) that have antimicrobial properties toward nearby pathogens. Mycoparasitism is another biocontrol mechanism of some yeasts which directly attack and retrieve nutrients from pathogenic fungi. Interestingly certain yeasts induce plant resistance to pathogens. These yeasts can stimulate the plant’s own defense mechanisms, enhancing resistance to various pathogens [9]. Hence, these applications reveal the versatility of yeasts in agricultural disease management.

Although the potential of biocontrol yeasts is evident, several challenges still exist. Limited understanding of the mechanisms emphasizes the need for deeper insights into the molecular and genetic bases of yeast–pathogen interactions [8]. Regulatory hurdles such as the approval process for biocontrol agents can be complex and time-consuming [8]. Ensuring consistent performance of biocontrol yeasts under diverse environmental conditions remains a challenge [10]. Therefore, despite their biocontrol evidence, yeast biocontrol agents are underrepresented in registered commercial products [8,11].

**Figure 1:**
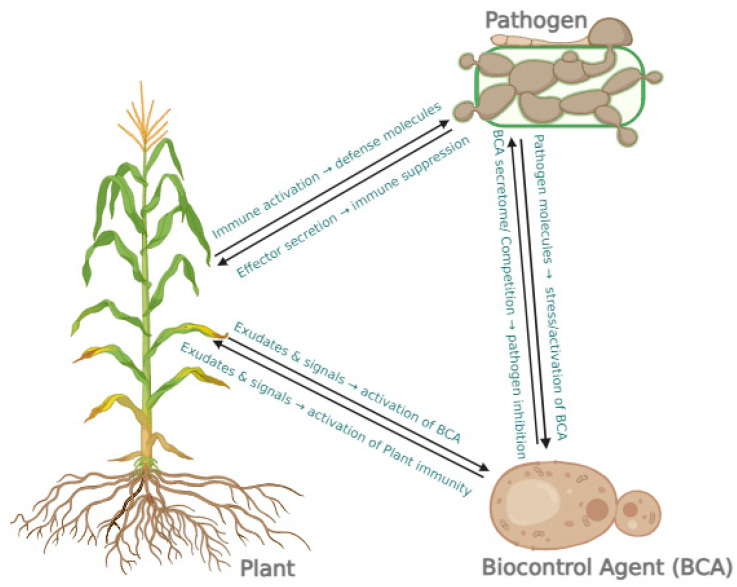
Tripartite interaction between plant, pathogen, and biocontrol agent (BCA). Created in https://BioRender.com.

Most of the BCAs are studied through pairwise interactions (plant + pathogen, pathogen + biocontrol agent, plant + biocontrol agent) or microbiome-wide approaches [12,13,14]. These pairwise studies show potential candidates in vitro, in planta, and in greenhouses under limited and controlled conditions. However, biocontrol ability highly varies in the large-scale agroecosystems [15]. This pairwise approach may cause many issues in practical applications. These issues on the practical applicability of BCA in agroecosystems emphasize the necessity of understanding tripartite relationships among biocontrol agents, plants, and pathogens (Figure 1). Several studies on other biocontrol agents with tripartite interactions demonstrate the necessity of understanding the behavior of each component in a tripartite system [8]. Moreover, comprehensive knowledge of this tripartite interaction is also required to identify the effective biocontrol strains to be used for efficient plant disease control [3]. Transcriptomic, proteomic, and metabolomic studies can provide better insights into these tripartite interactions at the molecular level [3,15]. Although the transcriptomic approach generates a vast amount of data, the study remains incomplete without the proteomic aspect, as it is directly responsible for cellular activity.

Therefore, the implications of proteomics, transcriptomics and comparative genomics regarding BCA interactions are gaining noteworthy attention. These techniques contribute to successful detection and characterization of major proteins and effectors that play key roles in inducing defense responses in plants against pathogens [16]. Nevertheless, understanding proteomics and effectors of BCAs can help identify novel determinants useful for developing novel biocontrol formulations with enhanced potential. Moreover, strain improvement using such determinants could also be achieved. In addition, a proteomic study of the pathogen in this interaction is of great interest, as it would provide insights into two aspects: the major factors contributing to the pathogenicity and targeting such factors for diminishing the pathogenicity [17]. Hence, it is important to understand the interactions of the whole microbiome to find successful identification of BCAs. This requires a broad understanding of interactions [18]. The current review investigates the gaps in the current knowledge on yeast-associated biocontrol and investigates the importance of considering tripartite interactions to explain the complex observations and missing links to establish a successful biocontrol product.

## 2. Established Roles of Yeasts in Biocontrol, Proteomic, and Metabolomic-Based Mechanisms in Yeast Biocontrol

Recent studies on biocontrol by yeast mainly focus on pairwise interactions of fungal–fungal or fungal–plant. Further, the potential was proven through in vitro and greenhouse methods. The main mechanisms contributing to yeast biocontrol are enzyme secretion, parasitism, volatile organic compounds (VOC) production, induction of systemic resistance, and competition for space and nutrients [8]. The widely used co-culture technique is a successful method for yeast secondary metabolite studies as it expresses certain inactive genes through microbial interaction [19].

Among other BCAs such as bacteria and filamentous fungi, yeasts have several advantages as they are genetically stable and relatively less pathogenic [10,20]. The most well-known yeast metabolites which are involved in biocontrol are the protein-like killer toxins [8], 2-methylenesuccinic acid, 2-propylacrylic acid, aureobasidins or liamocins, 3-amino-5-methylhexanoic acid, biphenyl-2,3-diol, and sinapaldehyde [19]. Recent studies revealed that yeasts could secrete more proteins with antifungal activity under in vitro conditions. However, it was difficult to quantify and validate the fungal inhibition under in vivo conditions. This suggests that the pathogen inhibition by yeasts involves synergistic or additive effects among different compounds and strategies [19]. Although many antagonistic yeasts are reported to work against fungal pathogens, the secondary metabolites involved in the antagonistic activity are not identified in most cases [19].

The exometabolomics together with classical co-culture studies by Fernandez-San Millan et al. [21] revealed that the antagonistic activities of yeasts are highly strain-specific. Further, the proteomic studies by Fernandez-San Millan et al. [21] revealed that two strains, *Pichia fermentans* Pf-31 and *P. terricola* Pt-36, have complex mechanisms of antagonism involving toxins (oxylipins, dipeptides, alkaloids) or antibiotics and biofilm formation. The *P. fermentans* Pf-31 and *Pichia terricola* Pt-36 showed the above effects against *B. cinerea* in apple. Interestingly, Fernandez-San Millan et al. [21] observed significant changes in the proteomes of Pf-31 and Pt-36 during the biocontrol process. Further, *Pichia fermentans* Pf-31 and *Pichia terricola* Pt-36 show remarkable biocontrol capabilities against *B. cinerea* on tomatoes and grapes [19,22,23,24]. Although yeasts can secrete diffusible compounds, they do not produce as many secondary metabolites as filamentous fungi or bacteria [8,25].

Interestingly, most of the yeasts can form biofilms. Attachment to fungal hyphae, biofilm formation, and wound colonization are some specific features of yeast. These properties are highly important in biocontrol in post-harvest applications [20,23]. It has been proposed that yeast cells in biofilms can destroy fungal pathogens by secretion of fungal cell-wall-degrading enzymes during the adhesion process [26]. Another hypothesis of the biocontrol ability of yeast is that the rapid growth and high density of yeast cells in fruit wounds contribute to the formation of biofilms that cover the entire wound area, allowing yeasts to adhere to and colonize surfaces more efficiently and increasing their resistance to stresses [27]. The changes in gene expression and increased catabolism through enhanced respiration in the presence of pathogenic entities indicate expansion of yeast growth and division, which could provide the compounds and energy needed for wound colonization [21]. Previous studies of proteomics in yeast strains suggested that all the proteins are strain specific. This further specifies that biocontrol responses are highly strain-specific, although general pathways such as genome expression, enhancement of energetic metabolism, oxidative stress responses, and cell envelope modifications are also implicated [21].

Several studies revealed the ability of yeast cells to elicit the host plant defense responses through activation of biochemical and structural defense systems against fungal pathogen development. Such actions involve reactive oxygen species (ROS)-scavenging mechanisms and increasing phenylpropanoids or pathogenesis-related proteins, although the reason for the observed changes is not well-understood [28]. Fernandez-San Millan et al. [21] observed the secretion of peroxiredoxin resulting from oxidative stress and thioredoxin domain-containing proteins by the biocontrol yeast in the presence of a host infected with the pathogen. However, enhancement of redox proteins could be related to an adaptive response of the cells to oxidative stress such as host wound and the pathogen action. The mechanisms of different yeasts are diverse, while each yeast strain possesses distinct characteristics and exhibits unique dominant mechanisms [29,30].

The above studies revealed that biocontrol properties are yeast strain specific. This indicates the presence of broader connections between the host, the pathogen, and the biocontrol agent. It is worthwhile to study the research studies explaining such complex interactions.

## 3. Tripartite Cross-Talks in Yeast Biocontrol Through *Blumeria graminis* f.sp. *hordei*—*Hordeum vulgare*—*Pseudozyma flocculosa* Tripartite System

The previous section discussed current knowledge and the evidence for yeast-based biocontrol. This mainly focused on the secretome studies of yeasts. However, secretome studies alone cannot define the yeast-based biocontrol mechanism, in which the tripartite interaction studies play a major role. This can be well explained through the *Blumeria graminis* f.sp. *hordei—Hordeum vulgare—Pseudozyma flocculosa* tripartite system. Early studies on in vitro assays of *Pseudozyma flocculosa* on biocontrol ability revealed that the mode of action of *Pseudozyma flocculosa* is through flocculosin-based antibiosis [31,32,33]. Later, Teichmann et al. [34] identified the gene cluster for regulating the synthesis of flocculosin. However, flocculosin nearly resembles ustilagic acid produced by *Ustilago maydis* and is controlled by a similar gene cluster [35,36]. Later, the taxonomic revisions identified *Pseudozyma* under Ustilaginales. Hence, the function of flocculosin and ustilagic acid should be similar as they are secreted from similar gene clusters and the studies revealed that ustilagic acid does not show functional similarity with flocculosin. This suggested that the mode of action of *Pseudozyma flocculosa* is not from flocculosin and its complex interactions in the presence of the pathogen. The genome comparison of closely related Ustilaginales and *Pseudozyma* revealed some 200 unique secreted candidate effector proteins and lytic enzymes, which could provide biocontrol ability against powdery mildew [37].

To address the complexity of the mode of action of *Pseudozyma flocculosa*, Laur et al. [37] studied the tripartite interactions among *Blumeria graminis* f.sp. *hordei* on *Hordeum vulgare* using *Pseudozyma flocculosa* by simultaneously analyzing transcriptomic responses, electron microscopic observations, and host phenotypic expressions. The studies on both phenotypic and transcriptomic responses revealed that 12 h post-inoculation (hpi), *B. graminis* conidial chains started to collapse. After 36 hpi, *P. flocculosa* started producing sporidia and had completely taken over *B. graminis* before the penetration began. The transcriptomic data shows low expression of flocculosin biosynthesis genes in planta, suggesting its limited role in biocontrol. Instead, during the interaction of *P. flocculosa* with *B. graminis*, a set of special secreted effector proteins (CSEPs), lytic enzymes, and transporters which are mainly for sugar and amino acid uptake, were strongly activated. As a result, *B. graminis* showed mixed gene activities such as a reduction in CSEPs from surface-growing (ectotrophic) structures, while those from haustoria were surprisingly increased, possibly as a survival attempt. The host *H. vulgare* reacted by increasing the expression of several defense-related genes, including pathogenesis-related (PR) proteins, peroxidases, and genes involved in oxidative stress responses. Simultaneously, genes involved in photosynthesis were turned down around 24 hpi but returned to normal by 36 h. These combined responses suggest that *P. flocculosa* works through non-toxic biological interference to control the pathogen and help the plant recover. Light and transmission electron microscopy observations supported the transcriptomic findings. At 12 hpi with *P. flocculosa*, barley haustoria appeared mostly intact, though some *B. graminis* ectotrophic hyphae had lost cytoplasmic content. By 24 hpi, visible degradation of surface conidia and hyphae was evident, and haustoria began showing signs of structural alteration, accompanied by a thickened extra-haustorial matrix. At 36 hpi, a clear layer of dead hyphae coated the leaf surface, and the remaining haustoria appeared collapsed or convoluted. These structural changes closely matched the timing of fungal suppression and effector activity observed in the transcriptomic data [37].

Interestingly the comparative genomics indicated remarkable similarities between *Pseudozyma flocculosa* and pathogenic smut fungi. In *Ustilago maydis,* the bE/bW heterodimer mating factor acts as a pathogenicity regulator. In *P. flocculosa,* bE/bW orthologs, downstream genes, or putative pheromone precursor and receptors were not transcribed [38]. This clearly indicates that *Pseudozyma flocculosa* lacks many pathogenicity-related effectors and evolved away from being a plant pathogen. Hence, *Pseudozyma flocculosa* is not a hidden pathogen, but a true biocontrol agent. Its non-pathogenic lifestyle is genetically encoded and not just conditional or environmental [37].

When the *B. graminis* transcriptomic profile following inoculation with *P. flocculosa* was considered, several transmembrane transporters, such as the haustorial-specific sugar transporter bgh00499 known to be involved in pathogenesis [39,40] were increased in expression as early as 12 hpi to a level that was maintained throughout the interaction. Considering the rapid growth of *P. flocculosa*, the transporter activity was increased in both fungi. This phenomenon could suggest the diversion of nutrients extracted by *B. graminis* from barley for the benefit of *P. flocculosa*. Transcriptome profiling of barley (*Hordeum vulgare*) during the interaction with *P. flocculosa* and *Blumeria graminis* revealed transient parasitism. The onset of fungal contact triggered strong activation of plant defense-related genes, accompanied by a temporary suppression of photosynthesis, which dipped notably at 24 hpi and recovered by 36 hpi [41]. This photosynthetic stress coincided with the upregulation of haustorial effectors from *B. graminis*, likely induced by *P. flocculosa* effectors, facilitating nutrient diversion from the plant to the biocontrol fungus [42]. The plant’s photosynthetic machinery thus became an indirect nutrient source for *P. flocculosa* through a brief “hyperbiotrophic” phase, terminating around 24 hpi with the collapse of *B. graminis*. This dynamic illustrates a rare, indirect form of plant parasitism mediated by the pathogen, positioning *P. flocculosa* as a transient epiphyte exploiting the mildew–plant interface [33,35,43].

## 4. Learning from Tripartite Models of *Trichoderma*

Tripartite interactions have been shown to be related to several other biocontrol agents. To understand the importance of knowing the tripartite interactions, it is worthwhile to study those interactions as case studies.

Avirulent, symbiotic, filamentous, saprophytic rhizosphere-inhabiting *Trichoderma* is a fungus with antagonistic properties toward many plant pathogens [44]. *Trichoderma* spp. represents 60% of the commercial biocontrol product market with prominent research investigations [45]. *Trichoderma* provides rhizosphere competence that facilitates root colonization and rapid establishment of BCA within the soil microbial community, enables biological control of plant pathogenic fungi through multiple mechanisms, and promotes plant growth. To understand the tripartite interactions, it is worth focusing on the existing knowledge on the effects of *Trichoderma* on plant pathogen and the effects of *Trichoderma* on plants [46].

Studies show *Trichoderma*, plant, and pathogen cross-talks achieved through chemical signaling, in which fungi produce chemical compounds that alter plant transcriptome, proteome, and metabolome [47]. Proteins, small RNAs, and different classes of secondary metabolites including VOCs have been documented to play different critical roles in *Trichoderma*–plant interactions [48,49]. *Trichoderma*-mediated plant growth regulation could either be the direct effect of released molecules on plants or an indirect effect of *Trichoderma*’s impact and modification of the surrounding environment, such as modifying soil microbiome or lowering soil pH making the macro- and micronutrients more available to plants [50,51]. The asymptomatic colonization of plant roots by *Trichoderma* shows several physical or biochemical responses, such as limiting the invading fungus to a few root cortical cell layers in plant roots, which includes reinforcing and modifying plant cell walls, as well as producing reactive oxygen species and antimicrobial secondary metabolites [52,53,54]. Further, the studies show that certain *Trichoderma* spp. can reduce the glycosyl hydrolases and peroxidases participating in plant defense. Studies show extended transcriptome changes in *Arabidopsis thaliana* associated with transient repression of the plant immune responses and downregulation of defense genes in the roots [55].

Plant hormones are essential in regulating complex and interconnected immune signaling networks, providing a huge potential for rapid response and adaptation to various conditions [56]. Microbially derived phytohormones can help plants withstand biotic and abiotic stress conditions. Several members of *Trichoderma* spp. can produce phytohormones (auxin, gibberellin), and the enzyme 1-aminocyclopropane-1-carboxylic acid (ACC) deaminase can regulate the level of plant ethylene [57]. By evaluating the relationship between the observed plant phenotype and hormonal variables, principal component analysis suggested a strong association between auxin induction and *Trichoderma*-induced plant growth promotion [58]. Hence, interfering with plant hormone homeostasis is a major way for *Trichoderma* spp. to interfere with plant physiology and improve plant fitness. In addition to plant hormones, *Trichoderma* spp. releases VOCs. Certain *Trichoderma* spp. and strains improved growth, plant biomass, chlorophyll content, and lateral root formation, while VOCs emitted by other species and strains had a negative effect [59]. Interestingly, 6-pentyl-2H-pyran-2-one (6PP) promoted plant growth and regulated root architecture in a specific and dose-dependent manner [60].

*Trichoderma* shows antibiosis and mycoparasitism or necrotrophic hyperparasitism on phytopathogenic fungi by producing an array of antibiotics and cell-wall-degrading enzymes such as chitinases, cellulases, and glucanases, that degrade polysaccharides, chlorophenolic compounds, hydrocarbons, and in some cases, even pesticide residues [61]. Further, *Trichoderma* spp. produces a wide range of biologically active secondary metabolites, showing antagonistic properties against phytopathogens [62,63]. Interestingly, *Trichoderma* spp. enhances the mobility of soil nutrients and increases the competition for the growth factors [64]. Apart from the effects on phytopathogens, *Trichoderma* spp. acts as a plant growth promoter by altering the plant physiology and by increasing the availability of plant growth factors [64].

The tripartite interaction between the host, *Trichoderma* spp., and pathogen is mainly the molecular changes during pathogen attack and/or plant responses [65]. Further, these interactions involve various defined factors, signaling molecules, and virulence and avirulence factors [65]. The proteomic studies support the understanding of the intricate network of interactions [66]. The diaminofluorescein fluorescence visualization techniques were used to understand tripartite network interactions of *Arabidopsis thaliana* infected with *Fusarium oxysporum* and BCA *Trichoderma asperelloides*. In plants, nitric oxide (NO) is important for defense against microbes. The above studies revealed that the drastic reduction in NO levels in plant roots during co-inoculation of *T. asperelloides* and *F. oxysporum* might explain failure of the pathogen to initiate disease symptoms in challenged plants [67].

Studies on *Arabidopsis thaliana* infected with leaf pathogen *Pseudomonas syringae* DC3000 (Pst) and roots treated with *Trichoderma asperelloides* led to a significant increase in expression of several Pst defense genes in the plant leaves, confirming systemic priming of plant defenses by *T. asperelloides* root treatment. Among the activation-altered genes, several marker genes for Et/JA pathways were upregulated; the lipid transfer protein, LTP4, encoding a pathogenicity-related protein, was highly upregulated. On the other hand, WRKY40, a transcription factor contributing to plant susceptibility to bacterial invasion, was downregulated [55]. Proteomic analysis was performed on the major changes in the *T. atroviride* proteome during double (*T. atroviride* + plant) or triple (*T. atroviride* + plant + pathogen) interactions with bean plants and the phytopathogens *B. cinerea* or *R. solani,* compared to the growth of *T. atroviride* alone [68]. A higher number of proteins were produced in the tripartite but not in the bipartite setup, indicating a well-adapted response of the antagonist to the pathogen presence during its interaction with the plant. Those proteins included a homolog of a 40 kDa heat shock protein, suggesting stress adaptation; chitin synthase involved in the synthesis of fungal cell wall, possibly to recover the damage caused by pathogens and plant enzymes; several cyclophilin family members playing various cellular roles; and a hydrophobin and ATP-binding cassette transporters performing various roles connected with survival, stress adaption, plant growth promotion, and defense [69,70]. Further, it revealed that activation of biocontrol-related genes and formation of special infection structures happened during seed germination [71]. In another tripartite interaction study between *T. atroviride*, tomato seedlings, and the phytopathogen *Phytophthora cinnamomi*, *T. atroviride* proved its ability to outgrow the oomycete in the competition for available space and nutrients from both the medium and root exudates, shielding the seedlings from root rot symptoms. In the same study, it was suggested that plant root exudates are likely to play a key role in biocontrol by inducing *T. atroviride* growth and sporulation assisting the BCA in the competition for space and nutrients [72]. These tripartite interaction studies on *Trichoderma* further suggest the importance of investigating the cross-talks between the host, pathogen, and biocontrol system. It is worth noting that *Trichoderma* BCA systems are well-studied under bipartite and tripartite interactions with their effectors and functions (Table 1).

## 5. Discussion

Searching for novel BCAs is crucial for modern agriculture and requires understanding of the biocontrol mechanisms. Several well-explained biocontrol mechanisms of selected biocontrol agents are presented in Table 1. Yeast shows high potential for being a successful BCA. Although many BCAs are available in the market, the representatives of yeast-based BCAs are low [11]. Among yeast-based BCAs available in the market, several products showed limited long-term commercial adoption. Products such as Aspire^®^ (*Candida oleophila*) and YieldPlus^®^ (*Cryptococcus albidus*) were among the first yeast BCAs marketed. However, they were withdrawn due to low and inconsistent efficacy under commercial conditions, difficulties in market development, and low profitability. Even though Nexy^®^ (*C. oleophila*) received EU registration in 2013, its market adoption remained limited [20]. Recent regulatory frameworks, such as the European Union (Regulation EC 1107/2009), require detailed mechanistic understanding, omics-level evidence, and robust field validation for biocontrol product approval [86]. This reflects a global trend where yeast BCAs are increasingly recognized as safe but require stronger scientific validation and innovative formulations to achieve sustainable adoption.

Most of the current research in yeast-based biocontrol is mainly focused on metabolites with biocontrol potential. This revealed a broad spectrum of yeasts with biocontrol ability and such research plays a crucial role in developing successful BCAs, by providing basic research grounds. Most of the studies are focused on bipartite interactions between BCA yeast and pathogens, BCA yeast and plants or pathogens and plants (which is more broadly studied). Even though this provides valuable details on disease implementation, disease response, and biocontrol ability, it is difficult to build the complete figure in mode of action without considering all three parties together. Referring to Table 1 and the above discussed data, yeast requires more studies to understand their biocontrol mechanisms.

In the current review, firstly, the existing knowledge on yeast bipartite interactions was discussed, and later, the tripartite interaction studies by Laur et al. [37] were discussed. Further, the studies on *Trichoderma* also discussed and emphasized the importance of knowing tripartite interactions, which shows the importance of studying either genomic or phenotypic response while considering Host–BCA–pathogen all together. Interestingly, these links can be studied either through excretome studies, or phenotypic expression. Fernandez-San Millan et al. [21] studied the secretion of oxidative stress response proteins to elucidate the BCA mode of action.

The study by Laur et al. [37] provides invaluable contribution to yeast-based BCA research. Their study shows how transcriptomics, comparative genomics, meticulous morphology, and stress response, separately, as well as all together, show the complex biocontrol mechanism behind the *Blumeria graminis* f.sp. *hordei—Hordeum vulgare using—Pseudozyma flocculosa* system. Furthermore, their study proved that the previously known flocculosin-based mode of action is misleading. Their study on parasitism mediated by the pathogen shows how much we should understand about the potential biocontrol agents to introduce sustainable and effective commercial biocontrol agents. The review clearly shows that yeasts have a wide range of biocontrol abilities. Although bipartite interactions show the biocontrol potential, the mode of action with the presence of a host can be more complex, and in certain instances, hidden life modes of BCA can be revealed [37].

*Trichoderma* is a well-established biocontrol agent and reviewing the tripartite interactions on *Trichoderma* biocontrol shows how important it is to study the host–pathogen–BCA system meticulously. *Arabidopsis thaliana*, *Trichoderma asperelloides*, and *Fusarium oxysporum*, show that *Trichoderma* primes the plant immune system not only by reducing nitric oxide accumulation in roots, which is a known infection marker, but also by activating ethylene and jasmonic acid-related defense genes in the leaves. This systemic immune priming is further supported by repression of negative regulators like WRKY40, reinforcing the role of *Trichoderma* as a holistic biocontrol partner [55].

Developing resilient and effective BCAs requires a multi-stage approach that integrates omics, phenotyping, and field validation. Omics technologies such as transcriptomics, proteomics, and metabolomics have become key to uncover BCA–plant–pathogen interactions. Transcriptomics and proteomics reveal gene and protein expression patterns in both BCAs and plants, enabling the identification of ISR-associated genes or secreted effectors, while metabolomics and VOC profiling uncover small molecules such as siderophores or phenylpropanoids that contribute to antagonism and plant defense priming [87]. Integrated multi-omics studies are increasingly applied to provide a holistic view of these complex interactions and to decipher molecular networks that shape biocontrol efficacy [88]. Mechanisms can be validated through phenotyping systems, including in vitro dual cultures and VOC suppression assays, as well as greenhouse trials which measure plant responses such as ROS levels, phenolic accumulation, and PR gene activation. Advances in imaging and sensor technologies, such as confocal microscopy and biosensors, now allow real-time monitoring of these interactions. The most promising strains must be tested under field conditions across different environments and seasons to evaluate disease suppression, plant growth benefits, and formulation performance. Further, strategic sampling enables translational omics to confirm whether pathways observed under controlled conditions are also engaged in the field. Combining phenotyping with field validation ensures the development of robust, mechanistically understood BCAs that can meet regulatory requirements and be practically deployed in agriculture.

Hence, the current review addresses the importance of studying tripartite interactions as a single system in BCA research. Further, the current research reviews the research strategies for tripartite interactions through proteomics, excretome analysis, transcriptomics, meticulous morphology, and stress responses to introduce sustainable high-performance BCAs.

## Figures and Tables

**Table 1:** Effectors in biocontrol agents and their function in biocontrol.

Biocontrol Agent	Effector/Protein (Type)	Function/Action	Target and Outcome	Reference
***Trichoderma* spp.**				
*Trichoderma virens*	Sm1 (cerato-platanin family)	Elicits systemic resistance; induces ROS and expression of a α-dioxygenase encoding genes	Enhanced resistance to fungal pathogens	[73]
*Trichoderma atroviride*	Epl1/cerato-platanin (CP)	Elicitor: monomeric CP induces ISR (induce the expression of a peroxidase)	Enhanced resistance to fungal pathogens	[74]
*Trichoderma harzianum*	Chitinases (Chi42/Chit42)	Mycoparasitism through degrading fungal cell walls; MAMPs for plant	Enhanced resistance to fungal pathogens	[75]
*Trichoderma longibrachiatum*	Type II hydrophobin	Antifungal activity; plant growth promotion (PGP); ISR through ROS, superoxide dismutase, oxylipin, phytoalexin, and pathogenesis-related protein formation or activity. Stimulates root formation and growth.	Enhanced resistance to fungal pathogens and growth promotion	[76]
*Trichoderma reesei*	Swollenin (expansin-like)	Alters lignocellulose-degrading enzyme system	Facilitates penetration, colonization, and ISR	[77]
*Trichoderma* spp.	Peptaibols (peptaibol peptides)	Antifungal activity; membrane lysis and cytoplasmic granulation	Direct pathogen inhibition and defense elicitation	[78]
*Trichoderma virens*	Sm2 (Sm1 paralog)	Root colonization, ISR modulation	Plant colonization and defense	[79]
*Trichoderma virens*	Secreted apoplastic proteins/CWDEs	Cell-wall hydrolysis, ROS modulation	Root apoplast modulation and enhanced colonization	[54]
**Yeasts**				
*Candida oleophila*	CoEXG1 (exo-β-1,3-glucanase)	Cell-wall-degrading enzyme secretes at wound sites	Postharvest pathogen inhibition on fruit	[80]
*Metschnikowia pulcherrima*	Pulcherrimin/pulcherriminic acid biosynthesis (PUL genes)	Induces antagonism through iron chelation	Pathogen suppression via iron starvation	[81]
*Aureobasidium pullulans*	Pullulan, CWDEs, Siderophores, Aureobasidins	Induces biofilm formation through adhesion and direct antagonism, causing competition	Widely used on fruit to reduce molds	[82]
*Metschnikowia pulcherrima*	Killer toxins/pulcherrimin-associated factors	Secretes antifungal proteins/toxins	Pathogen suppression	[83]
*Saccharomyces cerevisiae*	Killer toxins (K1, K2, K28 family)	Cation-selective pore formation; disrupts ionic homeostasis and inhibits growth; arrests DNA synthesis through K28	Pathogen inhibition	[84]
*Wickerhamomyces anomalus*	-	rapid colonization of the wounds; alters defense enzymes PPO, POD, APX, and SOD	Postharvest and in situ antagonism	[85]

## Data Availability

No new data were created or analyzed in this study. Data sharing is not applicable to this article.
